# Mechanistically informed non-invasive peripheral nerve stimulation for peripheral neuropathic pain: a randomised double-blind sham-controlled trial

**DOI:** 10.1186/s12967-021-03128-2

**Published:** 2021-11-06

**Authors:** Selina Johnson, Anne Marshall, Dyfrig Hughes, Emily Holmes, Florian Henrich, Turo Nurmikko, Manohar Sharma, Bernhard Frank, Paul Bassett, Andrew Marshall, Walter Magerl, Andreas Goebel

**Affiliations:** 1 The Pain Management Programme, Walton Centre NHS Foundation Trust, Lower Lane, Liverpool, L9 7LJ UK; 2 Pain Research Institute, Faculty of Health and Life Sciences, University of Liverpool, Liverpool, UK; 3 Centre for Health Economics and Medicines Evaluation (CHEME) Department, Bangor University, Bangor, Wales UK; 4 Department of Neurophysiology, Mannheim Centre for Translational Neurosciences, Medical Faculty Mannheim, Ruprecht Karls-University Heidelberg, Heidelberg, Germany; 5 Statsconsultancy Ltd, Amersham, UK

**Keywords:** Peripheral nerve stimulation, Peripheral neuropathic pain, Long term depression, Chronic pain, Low frequency

## Abstract

**Background:**

Induction of long-term synaptic depression (LTD) is proposed as a treatment mechanism for chronic pain but remains untested in clinical populations. Two interlinked studies; (1) A patient-assessor blinded, randomised, sham-controlled clinical trial and (2) an open-label mechanistic study, sought to examine therapeutic LTD for persons with chronic peripheral nerve injury pain.

**Methods:**

(1) Patients were randomised using a concealed, computer-generated schedule to either active or sham non-invasive low-frequency nerve stimulation (LFS), for 3 months (minimum 10 min/day). The primary outcome was average pain intensity (0–10 Likert scale) recorded over 1 week, at 3 months, compared between study groups. (2) On trial completion, consenting subjects entered a mechanistic study assessing somatosensory changes in response to LFS.

**Results:**

(1) 76 patients were randomised (38 per group), with 65 (31 active, 34 sham) included in the intention to treat analysis. The primary outcome was not significant, pain scores were 0.3 units lower in active group (95% CI − 1.0, 0.3; p = 0.30) giving an effect size of 0.19 (Cohen’s D). Two non-device related serious adverse events were reported. (2) In the mechanistic study (n = 19) primary outcomes of mechanical pain sensitivity (p = 0.006) and dynamic mechanical allodynia (p = 0.043) significantly improved indicating reduced mechanical hyperalgesia.

**Conclusions:**

Results from the RCT failed to reach significance. Results from the mechanistic study provide new evidence for effective induction of LTD in a clinical population. Taken together results add to mechanistic understanding of LTD and help inform future study design and approaches to treatment.

*Trial registration* ISRCTN53432663.

## Background

Neuropathic pain can arise either peripherally or centrally as a direct consequence of a lesion or disease affecting the somatosensory system. Classification of neuropathic pain syndromes, using quantitative sensory testing (QST) has defined patterns of loss or gain of function across sensory modalities (‘somatosensory profiles’) which may reflect underlying pain generating mechanisms [1]. Neuropathic pain arising from peripheral nerve injury is typically associated with positive sensory signs such as dynamic mechanical allodynia or pinprick hyperalgesia, features thought to reflect the sensitization of central pain pathways [2]. In rodent models of peripheral nerve injury which feature somatosensory profiles similar to those seen in nerve injury patients, abnormal impulses arising from peripheral nociceptors lead to enhanced pain-responsiveness of spinal cord dorsal-horn neurons [3]. This initiates an amplification of synaptic transmission in nociceptive pathways termed ‘nociceptive long-term potentiation’ (LTP), which is a pain-related variant of a ubiquitous mechanism of synaptic memory [4]. Experimental nociceptive LTP has been successfully established in humans by modelling the injury-related discharge through focal high-frequency electrical stimulation which facilitates long-lasting hypersensitivity specifically for mechanical stimuli [5, 6].

In rodent models, reversal of nerve-injury induced LTP is achieved through low frequency peripheral nerve stimulation which induces the counterbalancing process of ‘long term depression’ (LTD) where central nociceptive synaptic connections become actively weakened [7]; low frequency stimulation also reverses high-frequency stimulation-induced nociceptive LTP in uninjured animals [8], and healthy man [9]. Induction of LTD via low frequency stimulation should therefore aptly target persistent painful peripheral nerve injury through lowering enhanced gain in nociceptive pathways [10], however data about the operation of LTD and the effect of low frequency stimulation in these patients is lacking.

In clinical trials, surgical forms of peripheral nerve stimulation do not consistently utilise low frequency stimulation [11], whilst the design of low frequency transcutaneous electrical nerve stimulation (TENS) electrodes renders them less suitable for the induction of LTD at a tolerable stimulation level [12]. Low frequency stimulation through a transcutaneous-applied, small spherical electrode possible of inducing LTD has been explored in two uncontrolled trials but remains untested within controlled clinical trials [13, 14].

The current work sought to validate whether LTD can be induced within this clinical population employing an LFS technique and explore the potential efficacy of a non-invasive approach only to elicit LTD-related pain suppression. To do this, we conducted two interlinked studies. The research describes a parallel group, double blinded, sham-controlled randomised trial and an open label mechanistic study assessing psychophysical parameters pre and post low frequency stimulation. We hypothesised that for the open label mechanistic study a significant reduction (p =  < 0.05) in mechanical pain sensitivity and dynamic mechanical allodynia would be observed following low-frequency stimulation, and that within the RCT a significant difference (p =  < 0.05) in terms pain reduction in favour of the active treatment would be seen.

## Methods

### Study design

A single site parallel group, double blinded, sham randomised controlled trial (RCT) of external non-invasive peripheral electrical nerve stimulation (ENPENS), designed to assess the efficacy of ENPENS versus sham in patients with chronic pain following peripheral nerve injury. Patients were randomised to receive either active or control treatment and continued treatment for a period of 3 months (main treatment phase). As a further retention and recruitment aid, following completion of the main treatment phase patients were offered an optional cross over or treatment extension (3 months).

Screened subjects who met the inclusion/exclusion criteria either before or after completion of the extension/swap period were invited to participate in a further open label mechanistic study assessing psychophysical parameters pre and post low frequency stimulation to validate LTD as a working mechanism within a clinical sample.

### Participants

Suitable patients appearing to fulfil inclusion/exclusion criteria identified from the centre’s pain clinics received a pre-screening telephone assessment. Patients then attended a screening appointment where written informed consent was obtained by the study PI. Separate written consent was obtained for the mechanistic study.

Patients were eligible if they were aged 18 or older and had definite or probable pain post nerve injury of ≥ 12 months duration [15]. They experienced moderate to severe pain intensity (defined as an average of ≥ 5/10 on an 11-point (0–10) numerical rating scale (NRS) recorded daily over 1 week, but not dropping below 4 on any given day), in a localised area (distribution of one to two peripheral nerves to facilitate easily replicable independent stimulation), had brush stroke allodynia in that area (≥ 3/10 NRS) (prioritised by patients as an important clinical outcome) and had trialled first line pharmacotherapy (to ensure patients care was not disadvantaged via inclusion).

Patients were excluded if they had absolute numbness in the affected area, had known treatment contraindications, implantable devices for the same condition, unstable pain intensity of pain medications in the 6 weeks prior to the trial, had diagnosed psychiatric or mental health disorder or other health conditions/pain which in the opinion of the investigators would make the trial unsuitable, or were unable to comply with the study protocol. Additionally, patients were required to stop any medications that numb affected areas prior to the study to enable stimulation of the peripheral nerves. Lidocaine patches 2 weeks prior, Capsaicin treatments (both low-, and high concentration) 4 months prior to stimulation to allow nerve endings to grow back. Patients were requested not to commence any new medications/ treatments that may confuse evaluation of treatment efficacy. All prescribed and non-prescribed treatments were recorded throughout.

The criteria were the same for both studies with the exception that brushstroke allodynia (≥ 3/10 NRS) was not required for the mechanistic study. This study sought to demonstrate a reduction in mechanical hypersensitivity and although brushstroke allodynia is often a feature of this (exhibited by 15/19 patients) significant mechanical hypersensitivity can also exist without the presence of documented brushstroke allodynia [16].

### Randomisation

Once consented patients were randomised to either active or sham treatment. The study trial manager or PI randomised patients using an independent online randomisation service that employed a concealed 1:1 allocation schedule and varying block sizes of 2 and 4. Trial nurses and patients were blinded to treatment allocation and were informed that that the purpose of the trial was to compare two types of stimulation ‘Pen’ and ‘Flat’. They were further informed that efficacy was not related to strength of stimulus but rather determined by the electrical field (see active and sham device). Following randomisation assignment, trial nurses were provided with the appropriate stimulation device to issue to the patient. Randomisation was not employed for the mechanistic study.

### Setting of study

The study was conducted at a supra-regional UK national health services (NHS) neurology and neurosurgery hospital. The study was registered on the ISRCTN registry, registry number: ISRCTN53432663. The full protocol was published before initiation of the trial [17]. The trial was conducted in accordance with the original protocol. Data collected ended when all patients had completed the optional treatment extension/swap and was predetermined before commencement of the trial.

### Study objectives

The RCT primary objective was to establish whether ENPENS treatment versus sham treatment was effective in reducing pain for people with long-standing neuropathic pain following peripheral nerve injury, as measured by change in pain intensity following 3 months of treatment. Secondary objectives were to assess the benefits associated with treatment to other commonly affected areas such as quality of life, function, mood, self-efficacy (confidence to perform abilities in the presence of pain), reduction of allodynia in the areas of pain, and symptom report. The primary objective for the mechanistic open label study was to establish whether LFS was associated in reduction of enhanced pain-responsiveness in a clinical population, as measured by change in measured sensory features of clinically enhanced pain responsiveness. We hypothesised that within the RCT a significant difference (p =  < 0.05) in terms pain reduction, would be observed between groups in favour of the active treatment. Whilst for the open label mechanistic study a significant reduction (p =  < 0.05) in mechanical pain sensitivity and dynamic mechanical allodynia would be observed following low-frequency stimulation.

### Sample size

Sample size calculations were conservatively based on detection of a between-group difference of 1.5 following previous observational study data that had shown a mean treatment associated pain reduction of 2.8 units. The standard deviation of the outcome was assumed to be as per this data, 1.9 units [13]. A correlation of 0∙5 between the baseline and outcome pain scores was assumed (0.64 in observational study data). Therefore, based on a 5% significance level, 90% power, and assumed 30% attrition rate, it was calculated that 38 participants per group were required to show a difference of 1.5 units in the primary outcome between groups, further details are provided within the published protocol [17].

### Study procedures

#### Active and sham interventions

Low frequency nerve stimulation through a transcutaneous-applied, small spherical electrode that induces high current density is a long-established method to localize peripheral nerves for nerve blocks [18]. Active treatment was referred to as the ‘Pen device’ and utilised a transcutaneous peripheral nerve stimulation device with a pen shaped electrode (Xavant stimpod nms410, Pretoria, South Africa) with pre-set parameters of 2 Hz (Hz) and 1.0 ms (ms), and an adjustable stimulation strength of ≤ 30 milliamps (mA). The sham device was referred to as ‘the flat device’ and looked identical but used a flat 5 cm^2^ square adhesive electrode and parameters of 2 Hz, 0.1 ms, ≤ 6 mA (although appeared to allow 30 mA). The electrode and parameter combination created a perceivable, low current density not eliciting LTD [19]. Electrodes stimulated affected nerves, proximal to the focal area of pain and just outside of the identified area of allodynia. LTD requires delivery of ≥ 1200 pulses, requiring 10 min of treatment [5, 10]. To avoid unblinding due to felt or observed differences related to current density or LTD effect within training, the amplitude and stimulation time was limited (< 5ma and < 5 min) for both the active and sham devices. An independent physiotherapist with experience of stimulation began training by determining the point of stimulation and beginning stimulation for all patients but had no further patient contact- this was safeguard accurate nerve identification for stimulation and to maintain blinding of trial nurses.

#### Treatment dosage

Once able to demonstrate independent use of the device, patients were loaned a stimulator for 3 months, stimulating for a minimum of 10 min daily, at a mildly painful but not intolerable amplitude. Patients determined the amplitude, frequency, and timings of stimulation. Weekly telephone calls during the main treatment phase recorded treatment compliance and health care utilization.

#### Mechanistic study experimental paradigm

At baseline, the area of mechanical hyperalgesia was mapped using a pinprick stimulus of 256 mN along eight equally spaced tracks originating from the epicentre of pain. Then, a circular intervention array of 10 punctate electrodes (each 250 µm diameter), designed to preferentially activate small diameter epidermal nerve fibres (5), was placed over the epicentre of the pain. This electrode delivers electrical pulses using a constant current stimulator (Digitimer DS7A, UK). Baseline QST was then performed on areas directly adjacent the intervention array-electrode to obtain a comprehensive somatosensory profile, using the standardised German Research Network quantitative sensory testing (DFNS) protocol [20]. As part of this protocol, mechanical pain sensitivity (MPS) was assessed as the mean of the pain ratings in response to a geometric series of 8–512 mN calibrated pinpricks at factor 2 progression, and dynamic mechanical allodynia (DMA) as pain to gentle stroking touch (cotton wisp, QT-tip, soft brush); these MPS/DMA series were scheduled at the end of the QST procedure and were spaced by approximately 5 min between each. Then, electrical detection threshold was determined with single 2 ms duration electrical pulses of increasing strength through the array electrode using method of limits (felt/not felt). The subsequent LFS intervention consisted of a train of 2000 electrical, 2 ms duration, stimuli delivered through the array electrode at 1 Hz. Stimulation strength was initially 10 × EDT but, due to poor tolerability was reduced to 5 × EDT for the final 6 study patients. Directly after LFS stimulation, the hyperalgesic area was measured again and then MPS and DMA were determined as before.

### Outcomes

RCT outcomes were completed by patients and collected by trial nurses. Outcome questionnaires were scored, and all data entered onto a computer by a technical assistant independent to the trial. Data analysis was conducted by the unblinded statistician after data lock.

#### Primary outcome

The RCT primary study endpoint was the average 24 h pain intensity recorded daily on an 11-point (0–10, 0 = no pain & 10 = worst pain imaginable) numerical rating scale (NRS), averaged over the last 7 days of the three-month treatment phase. At least 1 daily score was required.

#### Mechanistic study outcomes

The co-primary outcomes were change in intensity of mechanical pain sensitivity and dynamic mechanical allodynia following LFS, normalised to baseline. The secondary outcome was the change in mechanical hyperalgesia area following LFS. Spontaneous post-test pain was not measured as this parameter was considered confounded by the lengthy examination protocol.

#### RCT secondary outcomes

Text within brackets indicates what each measure was intended to capture.

- Brief pain inventory interference subscale (BPI-I) (functional interference) [21].
- EuroQol EQ-5D-5L generic measure of health status. The EQ-5D-5L has two components, a summary index (utility), and the EQ visual analogue scale (EQ-VAS), (health related quality of life) [22].

#### RCT exploratory outcomes

- Hospital anxiety and depression scale (emotional function) [23].
- Pain self-efficacy questionnaire (perceived confidence to function despite pain) [24].
- Worst pain using BPI (range of pain intensity) [21].
- Dynamic mechanical allodynia determined by manual mapping (change in surface area of allodynia) [25].
- Neuropathic pain symptom inventory (NPSI) (quality of pain) [26].

Outcomes were recorded at baseline, treatment completion and on completion of the optional treatment extension/swap. Secondary and exploratory endpoints were scores following end of 3-month treatment phase. Treatment phase patient diaries captured daily pain intensities and treatment frequencies.

#### On study completion

- Patient perceived global impression of change (PGIC) [27].
- Telephone interview—qualitative exploration of ‘active’ treatment experience in a proportion of patients.
- Perception regarding treatment allocation. Patients were asked if they felt they had been assigned to a more or less effective stimulation.

#### Safety outcomes

At every patient contact, safety adverse events (SAEs) and serious adverse events (SARs) were recorded.

### RCT statistical analysis

The primary study analysis was intention to treat (ITT) based on all randomized, eligible patients with outcome measures available at the end of the study. For the primary outcome, the primary endpoint was compared between groups by Analysis of Covariance (ANCOVA) using baseline scores as the covariate. In a secondary analysis of the primary outcome (responder analysis) the proportion of patients in each arm that achieved distinct outcomes (≥ 2 points NRS, ≥ 30% and ≥ 50%) were computed. The minimally clinically important difference (MCID) was defined as ≥ 2 points [28]. No stratification variables were included in the primary analyses.

Equivalent methods were used for those secondary and exploratory outcomes measured on continuous scales. For analysis of ordinal outcomes, the Mann–Whitney test was used. MCID was calculated for all further outcomes as follows based on the available literature: BPI-I 2-point reduction, EQ-5D-5L VAS 11 points increase, utility 0.145 increase, HADS 4 points reduction plus movement between severity categories, PSEQ increase of 7 + points plus movement between severity categories, BPI worst pain 3-point decrease, 20% reduction in surface area of allodynia [25, 29–32]. NPSI was not included due to an absence of appropriate literature. Sensitivity analyses were performed for both the primary outcome, and for the secondary outcomes; multiple imputations (MI) were used to address the missing values, utilising approaches based on the multivariate normal distribution method [33]. Fisher’s exact test was additionally reported as part of secondary analysis to illustrate any association between groups in relation to outcome measures. For all outcomes statistical tests were two-sided, with p-values of p < 0.05 considered statistically significant. All analysis except for post-hoc analysis was prespecified and included as part of the published protocol prior to initiation of the trial. All data was analysed using Stata version 15.1 (Statacorp.2017) statistical software. Adverse events were summarised descriptively.

### Health economic analysis

Details of the healthcare resource use and cost analysis are described in the published protocol [17]. A pre-planned health economic analysis to estimate NHS perspective cost-effectiveness, will be reported separately.

### Mechanistic study statistical analysis

QST values (excluding paradoxical heat sensations and dynamic mechanical allodynia) were z-transformed using the mean and standard deviation (SD) of the healthy control normative data for age, gender, and body location [16]. Z-scores above zero indicate gain of function and below zero indicate loss of function. QST profiles were compared to normative healthy subject German Research Network quantitative sensory testing (DFNS) protocol data with mean = 0 and SD = 1, using non-paired t-tests [16]. Pain rating data and mechanical hyperalgesia area were Log10-transformed to obtain secondary normal distribution. For all NRS ratings a constant of 0.1 was added to avoid loss of any zero ratings [19]. Raw data were smoothed by 3-point averaging to reduce irregularity caused by swings of single rating values. Results are shown as mean and SEM of log10-transformed data. Data were analysed using Friedman test. All calculations were performed with SPSS 20 (IBMTM) and Excel 2010 (MicrosoftTM).

## Results

**Fig. 1:**
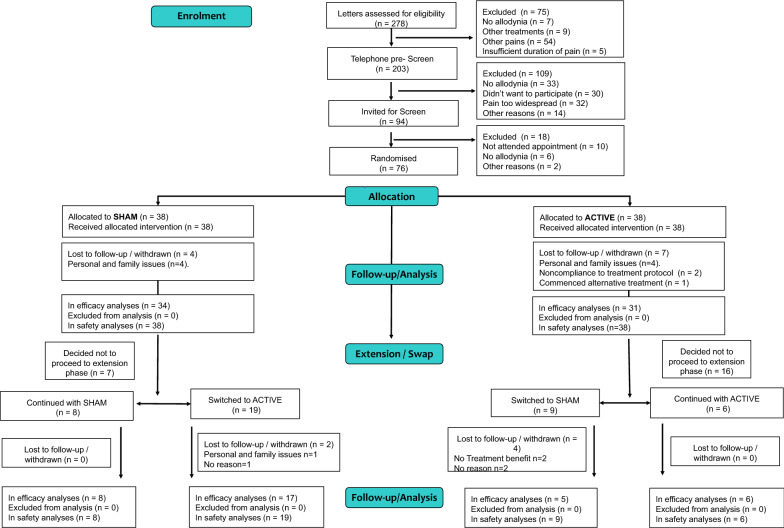
ENPENS trial profile (CONSORT diagram)

Within the RCT 278 patients were screened between January 26, 2017–July 11, 2019, 76 patients were randomised to treatment (38 per group), and 65/76 (86%) provided end of treatment outcomes and were included in the primary study efficacy analysis (31 active, 34 sham). On completion of the RCT treatment phase 42/65 participated in the optional treatment extension/ swap phase. Participant flow and reasons for withdrawal are detailed in Fig. 1. Enrolment stopped when the required sample size was obtained.

Seventeen of the 76 patients randomised entered the mechanistic study, along with four patients excluded from the RCT study due to low mechanical allodynia scores ≤ 3 numerical rating scale (NRS). Two of these 21 patients (both RCT participants) were unable to tolerate the electrical stimulation at × 5 EDT and withdrew, therefore 19 patients completed mechanistic testing. Fifteen of these had been randomised in the RCT study, of which five attended directly after the main treatment phase, ten after the extension/swap.

Patient demographic and baseline characteristics were broadly similar between the active and sham arms (Table 1).

**Table 1:** Patient demographics and baseline characteristics of Intention to treat analysis population

	Category	Active (n = 38)	Sham (n = 38)	All patients (n = 76)	Mechanistic study (n = 19)^#^
Age		47.3 ± 15.9	53.6 ± 11.2	50.4 ± 14.0	61.8 ± 14.9
Gender	Female	22 (58%)	18 (47%)	40 (53%)	9
	Male	16 (42%)	20 (53%)	36 (47%)	10
Duration Pain (months)		44 [27, 96]	48 [26, 72]	47 [27, 87]	61 [23,63]
Mechanism of Injury	Nerve entrapment	1 (3%)	2 (5%)	3 (4%)	2 (11%)
	Nerve injury				
	Surgery	29 (76%)	25 (66%)	54 (71%)	8 (42%)
	Other mech. Trauma	6 (16%)	7 (18%)	13 (17%)	6 (32%)
	Radiotherapy	2 (5%)	0 (0%)	2 (3%)	0 (0%)
	Medication	0 (0%)	1 (3%)	1 (1%)	0 (0%)
	Post-herpetic neuralgia	0 (0%)	3 (8%)	3 (4%)	2 (11%)
Number pain meds		1.6 ± 1.5	1.7 ± 1.4	1.7 ± 1.4	1.8 ± 1.1
Pain medications^+^	General pain meds	18 (58%)	24 (69%)	42 (64%)	3 (16%)
	NSAIDs	9 (29%)	13 (37%)	22 (33%)	2 (11%)
	Opioids	9 (29%)	7 (20%)	16 (24%)	7 (37%)
	Anti-Epileptics	15 (48%)	20 (57%)	35 (53%)	10 (53%)
	Anti-Depressants	16 (52%)	11 (31%)	27 (41%)	6 (32%)
	Muscle relaxants	1 (3%)	2 (6%)	3 (5%)	0 (0%)
Baseline assessments					
Primary	Pain in last 7 days	7.2 ± 1.2	7.5 ± 1.4	7.3 ± 1.3	7.42 ± 1.3
Variability pain*		0.85 ± 0.51	0.84 ± 0.44	0.85 ± 0.47	0.65 ± 0.55
Secondary	EQ VAS	51 ± 18	57 ± 25	54 ± 22	48 ± 27
	EQ-5D index	0.35 ± 0.23	0.34 ± 0.29	0.35 ± 0.26	0.27 ± 0.29
	BPI I	6.2 ± 1.9	6.4 ± 2.0	6.3 ± 1.9	6.3 ± 2.9
Exploratory	BPI W	8.4 ± 1.1	8.2 ± 1.4	8.3 ± 1.2	8.5 ± 0.9
	HADS anxiety	10.7 ± 4.3	10.4 ± 5.2	10.5 ± 4.8	9.8 ± 5.6
	HADS depression	9.3 ± 4.6	9.0 ± 4.5	9.1 ± 4.5	9.5 ± 5.7
	PESQ	24 ± 14	23 ± 14	24 ± 14	19.7 ± 13
	DMA mapped area	207 ± 192	175 ± 141	191 ± 168	204 ± 166
	NPSI total score	63 ± 15	61 ± 19	62 ± 15	53 ± 19

Summary statistics are mean ± standard deviation, median [inter-quartile range] or number (percentage). There were no significant differences in any baseline measures between active and sham groups

EQ VAS = EuroQol visual analogue score, EQ-5D Index = EQ-5D-5L index score (utility), BPI I = Brief pain inventory interference subscale, BPI W = Brief pain inventory worst pain intensity, HADS anxiety = Hospital anxiety scale anxiety subscale, HADS depression = Hospital anxiety scale depression subscale, PSEQ = Pain self-efficacy questionnaire, DMA mapped area = Dynamic allodynia mapped area, NPSI total = Neuropathic pain symptom inventory subscale total score

*Measured by the standard deviation of the baseline daily pain scores in the week prior to randomisation

^+^Patients could be on more than one type of pain medication. Percentage values may not add up to 100%

^#^ For mechanistic study the variables age, gender, duration of pain, mechanism of injury and information relating to medications were available for all 19 patients, but for all other outcomes, the numbers represent n = 15/19 available data sets

In the mechanistic study, full demographic data was not available for the four patients who had not participated in the RCT study, i.e., was available for 15/19 participants (Table 1). The patient age in the mechanistic study was significantly lower compared to RCT, t (91) = 3.13, p = 0.03, all other baseline variables were statistically comparable.

**Fig. 2:**
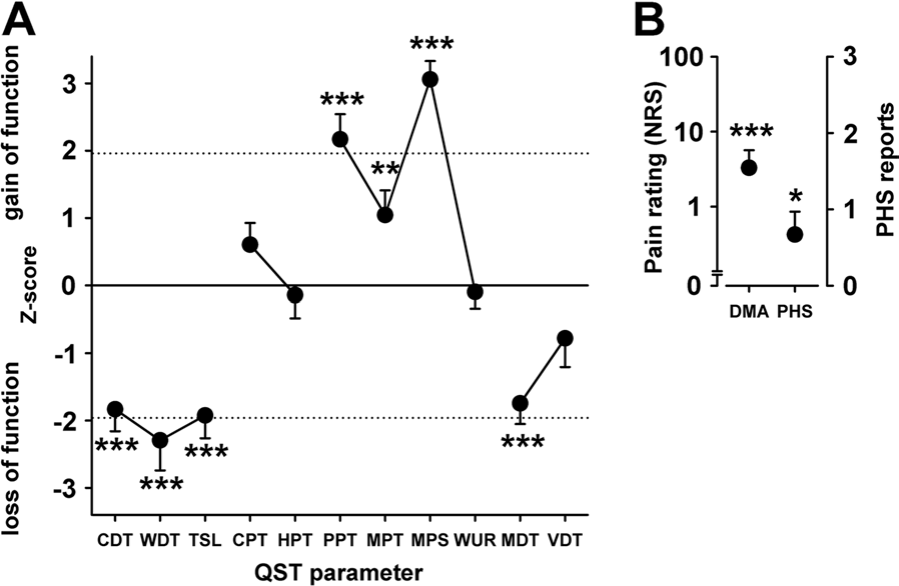
Baseline quantitative sensory testing (QST) profile for patients in the mechanistic study (n = 19). **A** CDT: cold detection threshold; WDT: warm detection threshold; TSL: thermal sensory limen; CPT: cold pain threshold; HPT: heat pain threshold; PPT: pressure pain threshold; MPT: mechanical pain threshold; MPS: mechanical pain sensitivity; WUR: wind up ratio; MDT: mechanical detection threshold; VDT: vibration detection threshold. Data are presented as mean z-scores for thermal and mechanical QST parameters. Values greater than 0 represents a gain-of-function. Data less than 0 represent a loss-of-function. Dotted lines indicate 95% confidence interval for normative German network on neuropathic pain (DFNS) data for healthy controls. **B** Baseline dynamic mechanical allodynia (DMA) and paradoxical heat sensations (PHS) in patients in the mechanistic study (n = 19). Data are mean numeric pain ratings for DMA on a logarithmic scale (0–100) and frequency of PHS (0–3). Any score for DMA is considered as abnormal. **A**, **B** p =  ≤ 0.05*, p =  ≤ 0.01**, p =  ≤ 0.001*** denotes this level of significance compared to normative DFNS reference data

Baseline quantitative sensory (QST) testing using DFNS protocol [20], was obtained for all patients within the mechanistic study (n = 19). QST profiles were compared to normative healthy subject DFNS data [16], and revealed a substantially abnormal QST pattern in patients shown in Fig. 2. Compared to normative data, the QST profiles indicated significant gain-in-function for mechanical pain sensitivity (p =  < 0.001), mechanical and pressure pain thresholds (p =  < 0.001), as well as dynamic mechanical allodynia (p =  < 0.001), but not thermal hyperalgesia. This mechanonociceptive gain contrasted to a significant loss of function in tactile and temperature detection thresholds (p =  < 0.001). Taken together these results emphasize that the mechanistic study successfully enrolled the targeted mechanical (not thermal) hyperalgesia pain phenotype of patients within these studies. This patient subgroup is most likely to harbour a central sensitization aspect, for which punctate hyperalgesia and dynamic mechanical allodynia are hallmark signs [1, 34, 35], associated with increased ongoing pain [36].

**Fig. 3:**
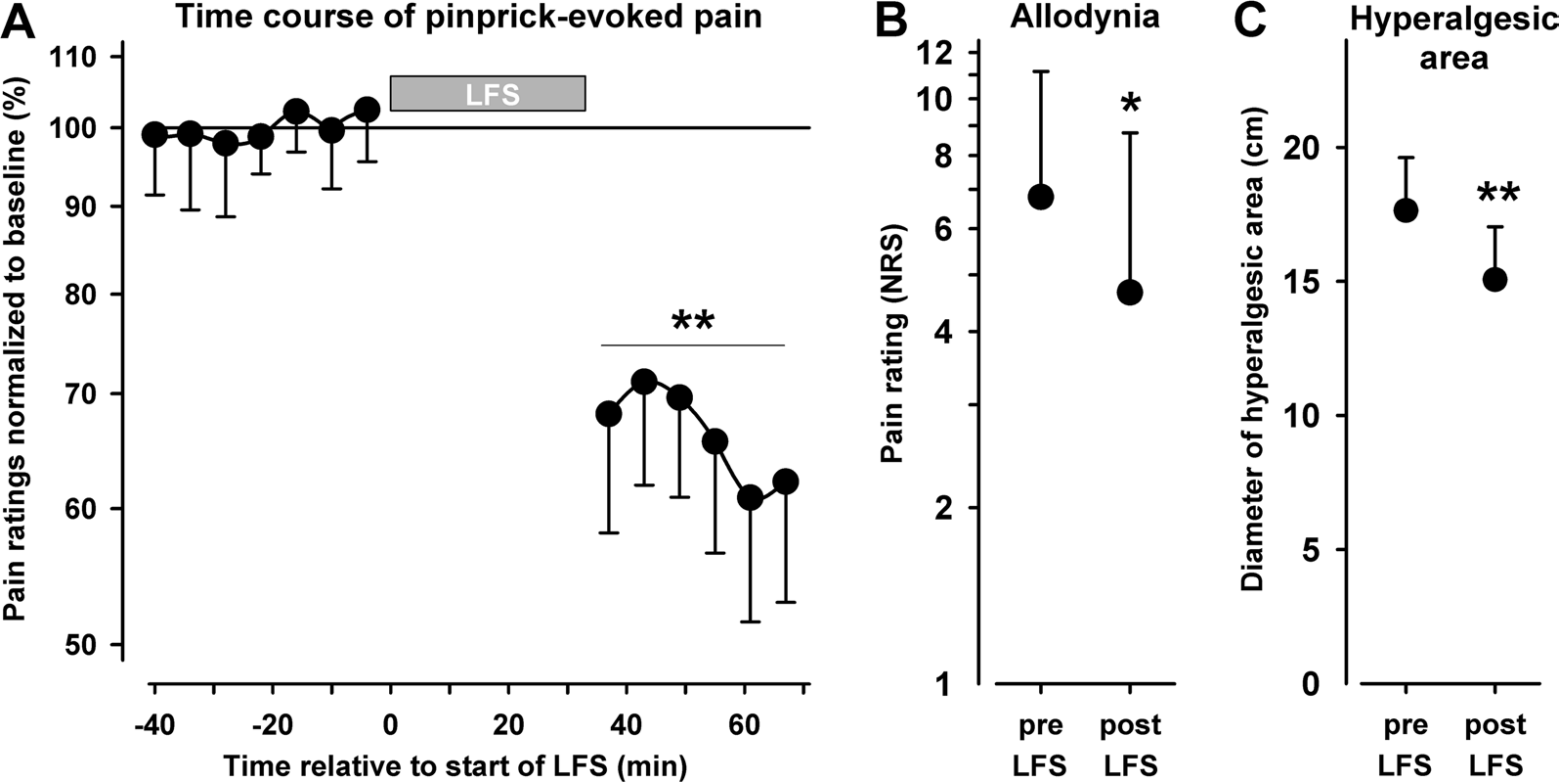
Mechanistic study outcomes: Suppression of mechanically evoked pain by LFS in the clinically affected area. **A** Mechanical pain sensitivity: A series of pain ratings in response to repeated sets of pinprick stimulations spaced approximately 5 min from each other, within the clinically affected area in patients who participated in the mechanistic study (n = 19). Data depict baseline ratings preceding low frequency stimulation (LFS), followed by LFS (no rating), and ratings to testing following LFS. **B** Dynamic mechanical allodynia (Pain to light touch): Means of pain ratings following stroking touch stimuli of the affected skin, before and after LFS in patients who exhibited DMA prior to LFS (n = 15). **C** Area of mechanical hyperalgesia: Means of diameter of area of mechanical hyperalgesia mapped by a punctate stimulus before and after LFS (n = 19). **A–C** Where *p ≤ 0.05 **p ≤ 0.01, comparing pre- to post-LFS pain ratings, Vertical error bars represent SEM

*Mechanistic study outcomes* The co-primary outcomes were change in intensity of mechanical pain sensitivity (MPS) and dynamic mechanical allodynia (DMA) pre and post LFS. MPS was assessed as the mean of the pain ratings in response to a geometric series of 8—512 mN calibrated pinpricks at factor 2 progression, and DMA as pain to gentle stroking touch (cotton wisp, QT-tip, soft brush). Pain ratings to pinprick stimuli were significantly reduced following LFS (average 30 min post-LFS = − 34.2%, p = 0.006, Fig. 3A). Correspondingly, in the 15 patients who exhibited DMA, this parameter reduced by 29.4% from 6.63 NRS to 4.66 NRS (log10 mean + SEM: 0.832 ± 0.215 vs. 0.669 ± 0.273; Friedman ANOVA p = 0.043, Fig. 3B). Ten of these patients experienced a reduction of allodynia, one remained unchanged, four reported a modest increase.

The secondary outcome was the change in mechanical hyperalgesia area following LFS. Area of mechanical hyperalgesia to pinprick was reduced by 22.1% from 300 to 233 cm^2^ following LFS, with a significant mean diameter reduction of (17.64 ± 1.98 vs 15.06 ± 1.98 cm; p = 0.008, Fig. 3C).

**Fig. 4:**
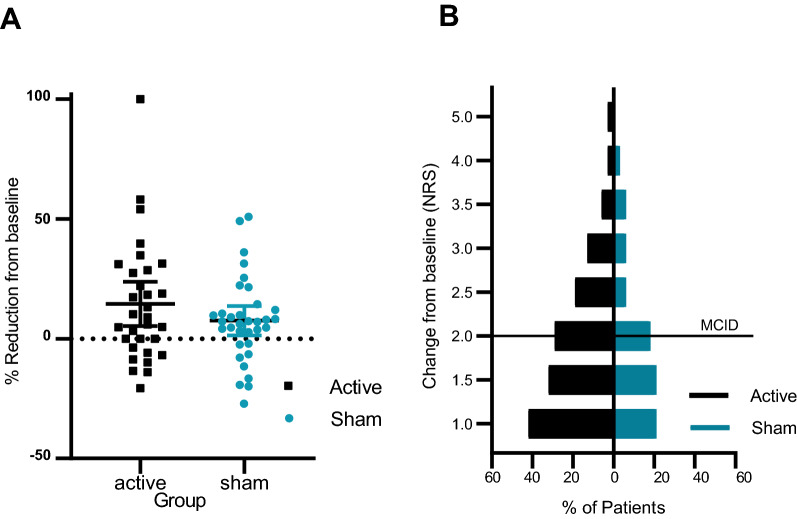
RCT Average spontaneous pain reduction at individual level, and in discrete improvement intervals. **A** Percentage reduction in the average NRS pain intensity from baseline to end of treatment. Negative scores denote worsening; **B** Percentage of patients achieving step-reductions in their average NRS scores from baseline to the end of treatment, where MCID denotes minimal clinical important difference of equal to 2; n = 31 active, n = 34 sham

*Clinical trial outcomes* The RCT primary outcome was the average 24 h pain intensity recorded on an 11-point (0–10, 0 = no pain & 10 = worst pain imaginable) numerical rating scale (NRS), recorded daily and averaged over the last 7 days of the three-month treatment phase, compared between study groups. Of the 76 patients, 60 provided all 7 daily pain intensity scores for the primary outcome in the last 7 days of treatment, 64 provided at least 3 daily scores and 11 (7 active, 4 sham) supplied none. The primary outcome was not statistically significant between treatment arms (Table 2). After accounting for baseline scores, pain scores were 0.3 units lower in the active group (95% CI -1.0, 0.3; p = 0.30) compared with sham, giving an effect size of 0.19 (Cohen’s D). In a secondary analysis of the primary outcome (responder analysis) the proportion of patients achieving minimally meaningful pain reduction (≥ 2 points) was 29% in the Active group, compared to 18% of the Sham group (Table 3 and Fig. 4). The treatment effect changed little when baseline pain was considered as an outcome to include the 11 patients without post randomization data.

**Table 2:** Study outcomes (intention to treat)

	Group	N	Baseline Mean ± SD	3 months Mean ± SD	Trt effect* Mean (95% CI)	P-value
Primary outcome						
Average NRS (over 7 days)	Active	31	7.1 ± 1.3	6.2 ± 1.9	− 0.3 (− 1.0, 0.3)	
	Sham	34	7.3 ± 1.4	6.7 ± 1.7	0	0.30
Secondary outcomes						
EQ VAS	Active	31	48 ± 18	61 ± 20	10 (0, 19)	
	Sham	34	57 ± 25	56 ± 24	0	**0**.**05**
EQ-5D Index	Active	31	0.36 ± 0.25	0.46 ± 0.29	0.04 (− 0.06, 0.14)	
	Sham	34	0.35 ± 0.29	0.41 ± 0.31	0	0.40
BPI I	Active	31	6.3 ± 1.9	4.9 ± 2.6	− 0.9 (− 1.7, 0.0)	
	Sham	34	6.3 ± 2.0	5.8 ± 2.3	0	0.06
Exploratory outcomes						
BPI worst pain	Active	31	8.4 ± 1.0	7.0 ± 1.9	− 0.8 (− 1.6, 0.1)	
	Sham	34	8.0 ± 3.0	7.4 ± 1.9	0	0.07
HADS anxiety	Active	31	11.0 ± 4.7	9.2 ± 5.1	− 0.9 (− 2.3, 0.5)	
	Sham	34	10.6 ± 5.1	9.7 ± 4.5	0	0.22
HADS depression	Active	31	9.4 ± 4.9	8.3 ± 4.9	− 1.1 (− 2.4, 0.3)	
	Sham	34	9.0 ± 4.5	9.0 ± 5.0	0	0.13
PESQ	Active	31	23 ± 13	28 ± 15	1 (− 2, 5)	
	Sham	34	24 ± 15	27 ± 16	0	0.46
DMA mapped area (cm^2^)	Active	31	211 ± 204	173 ± 215	− 74 (− 126, − 22)	
	Sham	34	180 ± 145	215 ± 202	0	**0**.**006**
NPSI total	Active	31	63 ± 15	52 ± 19	− 5 (− 12, 2)	
	Sham	34	59 ± 18	55 ± 16	0	0.13

N: the number of patients with both baseline and end of treatment outcomes; Average NRS: Average pain intensity; EQ VAS: EuroQol visual analogue score; EQ-5D Index: EQ-5D-5L index score (utility); BPI I: Brief pain inventory interference subscale; BPI W: Brief pain inventory worst pain intensity; HADS anxiety: Hospital anxiety and depression scale anxiety subscale; HADS depression: depression subscale; PSEQ: Pain self-efficacy questionnaire; DMA mapped area: Dynamic allodynia mapped area; NPSI total: Neuropathic pain symptom inventory subscale total score

* Trt effect = Treatment effect is difference in outcome between treatment groups, adjusted for outcome at baseline. All analyses using ANCOVA

** Total costs (mean and 95% confidence intervals generated from 1,000 bootstrap replications) relate to the 3-month periods prior to baseline and follow-up and exclude the cost of device.

**Table 3:** Minimally clinical important difference change (MCID) for outcomes

	Active		Sham		Fisher’s exact P-value
	N	n (%)	N	n (%)	
Primary outcomes					
≥ 2 points OR ≥ 30%*	31	9 (29%)	34	6 (18%)	0.131
Secondary outcomes					
EQ-VAS ≥ 11	31	12 (39%)	34	7 (21%)	0.061
EQ 5D-Index ≥ 0.145	31	14 (45%)	34	10 (29%)	0.088
BPI I ≥ 2	31	9 (29%)	34	6 (18%)	0.131
Exploratory outcomes					
BPI W ≥ 3*	31	8 (26%)	34	5 (15%)	0.134
HADS Anxiety ≥ 4**	31	8 (26%)	34	5 (15%)	0.134
HADS Depression ≥ 4 **	31	6 (19%)	34	2 (6%)	0.082
PSEQ ≥ 7 **	31	9 (29%)	34	6 (18%)	0.131
DMA mapped area ≥ ^***^	31	16 (52%)	34	10 (29%)	**0.039**
Total mean	31	33% (± 11)	34	19% (± 7.1)	**0.005¥**

EQ-VAS: EuroQol visual analogue score; EQ-5D Index: EQ-5D-5L index score (utility); BPI I: Brief pain inventory interference subscale; BPI W: Brief pain inventory worst pain intensity; HADS anxiety: Hospital anxiety scale anxiety subscale; HADS depression: Hospital anxiety scale depression subscale; PSEQ: Pain self-efficacy questionnaire; DMA mapped area: Dynamic allodynia mapped area;  ± standard deviation, ¥ Mann Whitney

* % change based on pain score at baseline

** Also met additional criteria of movement to different severity category

***  > 20% change based on area at baseline

A sensitivity analysis was performed using multiple imputation methods to impute data values for patients with missing primary outcome data at 3 months. Analysis involving multiple imputation [33], provided no evidence of a significant difference between groups (p = 0.22) and did not greatly differ from ITT analysis.

Secondary outcome comprised of three measures. The EuroQol EQ-5D-5L a generic measure of health status. The EQ-5D-5L has two components, a summary index (utility), and the EQ visual analogue scale (EQ-VAS) [22], both components were reported as separate scores. The brief pain inventory (BPI) interference subscale was also measured [21]. EQ-VAS scores were on average ten points higher (= better) in the active group (95% CI 0, 19; p = 0.05), and BPI interference subscale values were on average 0.9 points lower (= better) (95% CI – 1.7, 0.0; p = 0.06), no significant change was observed between groups for the ED-5D-5L summary index (p = 0.40) (Table 2).

Multiple imputation was used to impute data values for patients with missing data at 3 months, results suggested that there was no strong evidence of a difference between groups for any of the secondary outcomes and did not greatly differ from reported ITT results.

Dynamic mechanical allodynia (DMA) area determined by manual mapping surface was the only exploratory outcome demonstrating significant change between groups, being on average 74 cm^2^ lower within active group compared to sham (95% CI 22 to 126 cm^2^ lower; p = 0.006) (Table 2). More sham group patients demonstrated enlargement of the DMA area following treatment, (47%, n = 16 vs 29%, n = 9, p = 0.14 (chi square test)). Other outcomes which included the hospital anxiety and depression scale [23], the pain self-efficacy questionnaire (PSEQ) [24], worst pain as measured by the BPI [21], and the neuropathic pain symptom inventory [26], did not significantly change (Table 2).

Minimally important clinical differences were measured. The average percentage of patients achieving minimally important clinical difference in any given outcome domain was significantly higher in the active group compared to the sham group (33% ± 11 Vs 19% ± 7.1, p = 0.005, u = 10 Mann Whitney test), (Table 3).

In terms of patient’s assessment of outcome more patients within the active group patients felt they had been allocated a more effective vs less effective treatment (18 (64%) vs 10 (36%)) compared to sham group (11 (37%) vs 19 (63%), p = 0.04). 

A total of 203 adverse events (AEs) were reported which were comparable between groups (2.7 ± 2.0 per person in active vs 2.7 ± 1.9 per person in sham); of these two were serious adverse events, viral meningitis and shingles, considered not to be related to the study intervention. Three AEs, all concerning increased pain during stimulation were judged as definitely related to the device (active n = 2), whilst 10 AEs were evaluated as probably related (active = 3, including temporary bruising, redness).

During the Optional treatment extension/swap period NRS dropped by 1.0 points for those who extended active treatment (n = 5, 95% CI − 3.3, 1.3, p = 0.28). Those who switched from active to sham experienced a worsening of pain by 1.5 points (n = 5, 95% CI 0.4, 2.6, p = 0.02). There were no significant outcome changes for the other two subgroups following this extension period.

Post-hoc analysis using baseline disease characteristics, demographic information and outcome responses provided no evidence of a meaningful relationship between outcomes and variables. Post-hoc analysis using QST information in relation to responder and non-responder profiles additionally did not yield any meaningful outcome, on the background of small numbers and dilution from sham allocation in the trial.

**Fig. 5:**
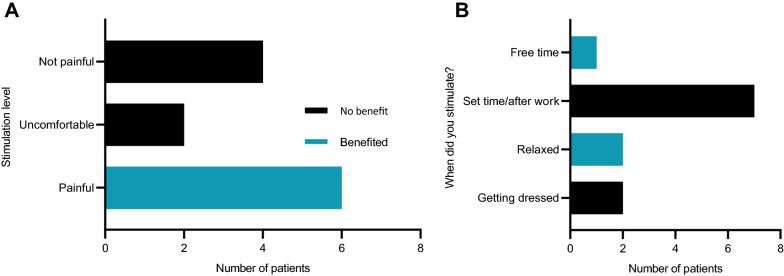
RCT Post hoc-Telephone Interview results. **A** Stimulation intensity versus perceived benefit. Represents the responses when patients were asked whether they perceived stimulation during the trial as painful. Patients also categorised into two groups according to whether they perceived the stimulator as beneficial or without benefit. **B** When did you use the device? Represents the responses to the question when they used their stimulator

Post hoc telephone interview was conducted to understanding patients experience of treatment. All patients who observed the stimulation as painful (n = 6/12) reported analgesic effect, but those who observed the treatment as either ‘uncomfortable’ or ‘not painful’ (n = 6/12) experienced no analgesic benefit. Patients indicated that the timing of their stimulator use was not influenced by their pain experiences, but more frequently conducted at a set daytime, Fig. 5. At trial start 7/12 patients expected to explore more invasive treatment options should stimulation prove unsuccessful.

## Discussion

We performed a combined RCT and mechanistic study to assess the effect of LFS via peripheral nerve stimulation treatment in patients with neuropathic pain after nerve injury. Mechanistic study results demonstrate consistent evidence for the induction of pain-LTD, and a corresponding significant reduction in stimulus-evoked pains in the affected areas, i.e., reduced hyperalgesia, which was further supported by the RCT outcomes.

External non-invasive low frequency peripheral nerve stimulation did not significantly reduce patients’ spontaneous pain, the primary RCT outcome. A trend toward a positive outcome across all outcomes in response to the active treatment rather than a definite effect supported by statistical change was observed. The intervention was well tolerated.

As mechanisms between neuropathic pains differ, we assessed QST profiles for all patients entering the mechanistic study at baseline and the results confirm that we had recruited the targeted group of patients who displayed a mechanical hyperalgesia sensory profile (Fig. 2); this profile is thought to reflect prominent central sensitisation due to nociceptive LTP [1, 3, 4, 34, 35]. The results from the mechanistic study confirm that LFS effected clear reductions of the patients’ stimulus-evoked pains, indicating the effective operation of pain-LTD. This is supported by exploratory RCT outcomes that showed significant reduction in dynamic mechanical allodynia (p = 0.006). Improvement in pinprick analgesia was larger than DMA reduction in the mechanistic study (Fig. 3), in keeping with current evidence [5].

The underpinning mechanism for pain-LTD is an intermediate rise of postsynaptic calcium concentration in nociceptive dorsal horn neurons (> 1 µM/L) inducing long-lasting depotentiation of synaptic transmission via increased phosphatase activity, diminishing postsynaptic LTP maintenance mechanisms. Under conditions of LTD only a small volume of calcium permeates into the post synaptic cell, which reduces the availability of receptors by translocation and consequently weakens synaptic efficacy [37]. The principle, that LFS can induce pain-related LTD to treat established pain-LTP, had earlier been established in healthy volunteers [19]. This study is the first to confirm that LTD can be induced via LFS within a neuropathic pain clinical population, supporting the rationale for the therapeutic approach.

Unlike in preliminary open-labelled studies using the same stimulator device [13, 14], reduction of stimulus-evoked pain was not paralleled by significantly reduced spontaneous pain within the RCT. Although it is possible that LFS does not reduce spontaneous pain intensity, several lines of evidence suggest that this outcome may reflect both sub-optimal stimulation intensity and frequency. In the mechanistic study, LTD was successfully induced by painful LFS stimulation at 5 × EDT, whereas in the RCT patients determined stimulus strength ad libitum. Post-hoc telephone interviews illustrated that only patients who experienced the stimulation in the active arm as painful, but none of the other patients found the treatment beneficial. Painfulness of an electrical stimulus is a function of the stimulus strength [38], and evidence from volunteer studies indicates that a just noticeably painful stimulus strength of 2–10 × detection threshold is effective while weaker or stronger stimuli are either less effective or completely ineffective [5, 19]. It is likely, therefore, that for maximal effect, clinically the LFS intensity should be mildly painful and that adherence to initial instructions was not consistently followed by the RCT participants. Furthermore, post-hoc telephone interviews indicated that most patients used the stimulator at a set daytime regardless of their actual pain experiences. This would suggest that patients viewed treatment as something that acted over a 24-h period (like a drug). As the duration of the LTD effect is unlikely to exceed a few hours [13], patients may therefore have missed out the potential of using the treatment to target either spontaneous- or activity-induced pain increases that would be variable during the day. Patient education in relation to treatment mechanisms, required stimulation strength and timing of treatment may therefore optimize clinical benefit by individually tailoring the stimulation but require further evaluation.

It is, however, also possible that reduction in stimulus-evoked pain in some patients does not fully translate into reduction of their spontaneous pain. Although we were recently able to show in a genotype–phenotype association study that more punctate hyperalgesia predicted stronger ongoing pain the correlation still remained low [36]. A disconnect between spontaneous and stimulus evoked neuropathic pain has been illustrated elsewhere [39, 40]. Topical lidocaine has been shown to reduce DMA for up to 3 months in patients with neuropathic pain after knee surgery, without global pain reduction [39]; and mechanical allodynia has been demonstrated in the absence of spontaneous pain [40]. Any partial disconnect between reductions in stimulus-evoked and spontaneous pains may reflect the multiplicity of mechanisms and the heterogeneous nature of neuropathic pain and treatments even within stratified sensory subgroups [39]. We demonstrated in both studies reduction in enhanced pain sensitivity following low-frequency nerve stimulation. This therefore appears to be a consistent effect associated with low frequency nerve stimulation. A decrease in marked skin sensitivity represents an important change in pain presentation and may still be considered/desired as a meaningful effect for patients in the absence of spontaneous pain reduction.

Pain is a subjective experience and therefore how it is evaluated will depend on contextual factors such as emotional wellbeing, physical functioning, and social occupational factors [24, 28, 31]. Due to the context of outcome evaluation in both studies contextual outcomes will have been of greater influence in the clinical trial. This is of relevance when we consider changes in pain evaluation was not significant in the clinical trial whilst significant change was seen for non-contextual QST outcomes in the mechanistic study. It is difficult for studies to measure relevant contextual factors and whilst the exploratory measures within the clinical trial in part attempted to do this, we were unable to find any association between exploratory measures and pain outcomes.

The current work was informed by clear mechanistic objectives and presents a novel approach of combining clinical and mechanistic study designs to evaluate and validate therapeutic potential. The RCT included successful recruitment and retention of a very specific group of patients with neuropathic pain, verified by QST, in consenting patients, which also validates the clinical recruitment criteria used. Patient selection could have been further strengthened by the inclusion of neuropathic pain specific screening questionnaires. The development and use of a true sham intervention with some perceivable but in-efficient stimulation parameters is a further strength—the lack of credible sham intervention has previously been noted as a limitation in neuromodulation trials [41, 42]. The study achieved a high level of patient adherence resulting in high data quality reducing uncertainty, and active and comparator groups were well balanced.

A limitation of the mechanistic study was the exclusion of a measure of spontaneous pain. Spontaneous post-test pain has a strong subjective component whilst other QST measures provide more objective assessments of pain processing. Given the subjective nature of this measure and the lengthy testing protocol (4 h) within the mechanistic study, we considered this measure would be too confounded to provide useful data. Ways of overcoming this should be considered in future study designs. Reduction in spontaneous pain intensity was not observed in the RCT and therefore LFS may not reduce spontaneous pain. We also recognise that suboptimal stimulation in the RCT may have influenced outcomes. The RCT protocol allowed patients to continue treatment without further advice or corrections after initial training. Post-hoc interviews have highlighted that this approach might have diminished the intervention’s effectiveness. Furthermore, within the RCT patients stimulated just outside the area of pain whilst in the mechanistic study stimulation was performed within the area of pain. Stimulation outside the area of the parent axon would have required deeper depth of penetration to drive axonal transmission along the nerve to the area of pain. Evaluation of stimulator use was limited as devices were not equipped with a system to monitor compliance or settings. Additionally, stimulation parameters were not recorded as part of the study. In normal practice, suboptimal stimulation may be improved with education relating to stimulation electrode positioning and coverage, but due to blinding this was not possible in the RCT.

A further limitation to the RCT is the inclusion of patients who may not have benefited from treatment such as patients on high dose opioids, psychological co-morbidities or potentially unresponsive to single nerve stimulation pain conditions which could have inadvertently underpowered the study. For example, we included patients with postherpetic neuralgia (PHN, n = 3 sham), radiotherapy induced nerve pain (n = 2, active) and medication induced nerve pain (n = 1, sham). PHN is typically associated with multisegmented dorsal horn atrophy which in turn limits the ability to correctly target a singular peripheral nerve with stimulation. Whilst radiotherapy and medication induced nerve pain is rarely confined to the distribution of one nerve. The three patients included with the later indications all experienced hand pain and only reported pain in the radial or ulnar nerve territory following said treatments; of note none of these patients had participated in the mechanistic study.

A further RCT limitation was incomplete availability of QST data for all patients included in the RCT. Broad availability of QST information might aid better understanding of responder and non-responder profiles supporting the stratification of patients in future studies. Patients were asked to indicate if they felt they had been assigned to a more or less effective intervention. Within the active group, most patients correctly identified that they had received the more effective treatment which may reflect that more patients experienced treatment benefit. A formal assessment of blinding however was not included during initial training, i.e., *before* delivery of any effective stimulation and therefore we cannot confidently exclude any unaccounted-for unblinding effect and its impact on outcome.

## Conclusions

Neuropathic pain can persist long after the initial cause has resolved and is often severely debilitating [15], affecting patients’ physical, economic, and emotional wellbeing and current treatment options have only modest outcomes [43, 44]. Results from our mechanistic study provide novel evidence for effective induction of long-term depression in a clinical population, an important requisite step to advance research and therapy in this area. Evidence from the RCT for the primary outcome of pain reduction failed to reach significance. Taken together results demonstrate the potential of low frequency stimulation and the need for further application enhancement, which will help inform future study design, and approaches to treatment. Low frequency stimulation is well tolerated, comparing favorably with drug treatments for the same patient group [44].

## Data Availability

Requests for patient-level data and statistical code should be made to the corresponding authors and will be considered by members of the study teams, including the study PIs and the sponsor sites neuroscience research unit (NRU) on a case-by-case basis. Access will be provided to researchers after the proposal has been reviewed and agreed by the sponsor sites data sharing committee, and the trusts IG department, beginning 3 months and ending 5 years following article publication. The data will not contain any direct identifiers, we will minimise indirect identifiers and remove free text data to minimise the risk of identification.

## Abbreviations

RCT: Randomised controlled trial
LTD: Long term depression
LFS: Low frequency stimulation
LTP: Long term potentiation
EDT: Electrical detection threshold
DMA: Dynamic mechanical allodynia
ENPENS: External non-invasive electrical nerve stimulation
TENS: Transcutaneous electrical nerve stimulation
mA: Milli amps
ms: Milli seconds
Hz: Hertz
QST: Quantitative sensory testing
PGIC: Patient global impression of change
SARs: Serious adverse events
SAEs: Safety related adverse events
NPSI: Neuropathic pain symptom inventory
MCID: Minimally clinical important difference
NHS: National Health Service
SEM: Standard error mean
DFNS: German research network
ANOVA: Analysis of covariance
MPS: Mechanical pain sensitivity
ITT: Intention to treat
EQ-VAS: Euroqual 5D-5L visual analogue health rating scale
BPI: Brief inventory scale
NRS: Numerical rating scale
PHN: Postherpetic neuralgia
