# Correction: Mechanistically informed non-invasive peripheral nerve stimulation for peripheral neuropathic pain: a randomised double-blind sham-controlled trial

**DOI:** 10.1186/s12967-023-04131-5

**Published:** 2023-04-29

**Authors:** Selina Johnson, Anne Marshall, Dyfrig Hughes, Emily Holmes, Florian Henrich, Turo Nurmikko, Manohar Sharma, Bernhard Frank, Paul Bassett, Andrew Marshall, Walter Magerl, Andreas Goebel

**Affiliations:** 1 The Pain Management Programme, Walton Centre NHS Foundation Trust, Lower Lane, Liverpool, L9 7LJ UK; 2 Pain Research Institute, Faculty of Health and Life Sciences, University of Liverpool, Liverpool, UK; 3 Centre for Health Economics and Medicines Evaluation (CHEME) Department, Bangor University, Bangor, Wales UK; 4 Department of Neurophysiology, Mannheim Centre for Translational Neurosciences, Medical Faculty Mannheim, Ruprecht Karls-University Heidelberg, Heidelberg, Germany; 5 Statsconsultancy Ltd, Amersham, UK

**Correction: J Transl Med (2021) 19:458** 10.1186/s12967-021-03128-2

Following publication of the original article [1], we have been notified that there was incorrectly mentioned device in the text body of the article (Methods’ section). It should be as follows:

Xavant stimpod nms410, Pretoria, South Africa
